# Experiences with Individual Placement and Support and employment – a qualitative study among clients and employment specialists

**DOI:** 10.1186/s12888-021-03178-2

**Published:** 2021-04-07

**Authors:** Miljana Vukadin, Frederieke G. Schaafsma, Harry W. C. Michon, Marianne de Maaker-Berkhof, Johannes R. Anema

**Affiliations:** 1 Amsterdam UMC, Vrije Universiteit Amsterdam, Department of Public and Occupational Health, Amsterdam Public Health research institute, Van der Boechorststraat 7, NL-1081 BT Amsterdam, The Netherlands; 2 Research Center for Insurance Medicine: Collaboration Between AMC– UMCG – UWV – VUmc, Amsterdam, The Netherlands; 3 Trimbos Institute, The Netherlands Institute of Mental Health and Addiction, Da Costakade 45, 3521 VS Utrecht, The Netherlands; 4 Movisie, Churchilllaan 11, 3527 BG Utrecht, The Netherlands

**Keywords:** Mental illness, Supported employment, Implementation, Employment, Barriers, Facilitators

## Abstract

**Background:**

Individual Placement and Support (IPS) is an evidence-based, effective approach to help people with severe mental illness (SMI) achieve competitive employment. The aim of the present study is to explore experiences with Individual Placement and Support using a multifaceted implementation strategy (IPS + MIS), and competitive employment. The goal of this strategy was to improve IPS implementation by enhancing collaboration between mental health care and vocational rehabilitation stakeholders, and realizing a secured IPS funding with a ‘pay for performance’ element.

**Methods:**

A qualitative, exploratory study was performed using semi-structured interviews with IPS clients (*n* = 10) and two focus groups with IPS employment specialists (*n* = 7 and *n* = 8) to collect rich information about their experiences with IPS + MIS and competitive employment. Thematic content analysis was used to analyse the data.

**Results:**

Themes related to experiences with IPS and the multifaceted implementation strategy were identified, including the importance of discussing the client’s motivation and motives to work, facilitators and barriers to obtaining and maintaining employment, facilitators to collaboration between stakeholders, barriers to benefits counselling, organizational barriers to IPS execution and collaboration between stakeholders, financial barriers to IPS execution and experiences with the pay for performance element.

**Conclusions:**

Although the multifaceted implementation strategy seems to contribute to an improved IPS implementation, the barriers identified in this study suggest that further steps are necessary to promote IPS execution and to help people with SMI obtain and maintain competitive employment.

**Supplementary Information:**

The online version contains supplementary material available at 10.1186/s12888-021-03178-2.

## Background

Employment is important for the recovery of people with severe mental illness (SMI) [1–5]. However, their employment rates are low [6, 7] and they often rely on social assistance or disability benefits [4]. Although obtaining and maintaining employment is difficult for many people with SMI, most of them want to work [8–10].

In the Netherlands, a widely used definition of SMI is: a psychiatric disorder that requires care or treatment, for which coordinated care from professional care providers in care networks is indicated to realize the treatment plan. The disorder is accompanied with serious impairments in social and/ or societal functioning and is persistent over time; the impairment is the cause and result of the psychiatric disorder [11]. In the Netherlands, 60% of the people with SMI have a psychotic disorder, such as schizophrenia, affective or organic psychosis; 40% of them have other diagnoses, such as autism, a severe depression or a personality disorder [11].

Individual Placement and Support (IPS) supported employment is an evidence-based, effective approach to help people with SMI obtain and maintain competitive employment [12]. IPS employment specialists offer this service and play a key role in this approach. These practitioners are guided by the following eight IPS principles:

1) Competitive employment: IPS services aim to get people into competitive employment. Competitive employment is defined as work in the community that anyone can apply for and pays at least minimum wage.

2) Zero exclusion: IPS employment specialists help anyone who expresses a desire to work; for example, people are not excluded on the bases of diagnoses, symptoms or disabilities.

3) Integration of mental health and employment services: IPS employment specialists attach to one or two mental health treatment teams (i.e. mental health care practitioners, such as case managers and psychiatrists). They have frequent meetings with their team(s) in which they discuss their caseload.

4) Client’s preferences: IPS services are based on client’s preferences and choices rather than on the employment specialist’s and mental health care provider’s judgments.

5) Personalized benefits counselling: IPS employment specialists help clients obtain personalized, understandable, and accurate information about how work may affect their benefits.

6) Rapid job search: IPS services focus on rapid job search rather than pre-employment assessments, training and counselling to help clients obtain employment.

7) Systematic job development: IPS employment specialists develop and maintain relationships with various employers, building an employer network. They systematically visit employers, who are selected based on the client’s preferences, to learn about their business needs and hiring preferences.

8) Time-unlimited and individualised follow-along support: IPS employment specialists provide time-unlimited, individualised support for as long as the client wants and needs it [13].

Although the effectiveness of IPS regarding obtaining and maintaining employment in people with SMI is well established [14–16], implementation of this approach has been challenging [17–20]. Poor collaboration among stakeholders from organizations involved in IPS (i.e. mental health agencies, benefits agencies and health insurance companies), and inadequate funding are important barriers to IPS implementation [6, 18–23]. In the Netherlands, for example, when IPS was first introduced, there were no formal agreements related to the collaboration between the stakeholders involved in IPS and funding of IPS. More specifically, there were no structural meetings between these stakeholders and IPS services were usually partly funded by health insurance companies or one of the benefit agencies, depending on the mental health agency. In practice, it was rather unclear which part was funded by which organization during an IPS trajectory.

To improve IPS implementation in the Netherlands, stakeholders from a mental health agency, the Dutch Social Security Institute: the Institute for Employee Benefits Schemes (UWV), the municipality of Amsterdam, and a health insurance company started to collaborate since 2014; in 2015 stakeholders from another mental health agency also joined. This collaboration included a multifaceted implementation strategy, comprising an organizational and a financial strategy.

The organizational implementation strategy consisted of regular meetings between the different stakeholders involved at two levels:

1) At the management level, there were regular meetings between decision makers who were considered key leaders within their organization. Their main goal was to ensure IPS sustainment.

2) At the practitioner level, there were regular meetings between IPS employment specialists and several vocational rehabilitation professionals within the benefits agencies involved. These practitioners discussed whether new IPS applicants qualified for funding, the progress of the current IPS clients, and any questions related to the clients’ benefits. Their main goal was to organize the IPS funding for new clients, and to provide optimal and improved benefits counselling as compared to usual IPS practice [19].

The financial implementation strategy consisted of secured IPS funding with a ‘pay for performance’ element, rewarding the mental health agency with extra payments for placing an IPS client in a competitive job. The duration and amount of the funding (excluding intake and job coaching) depended on the type of benefits the client received. The duration of the IPS funding for clients who received social assistance benefits from the municipality was limited to 18 months in the first year of the collaboration and thereafter to 24 months; for clients who received disability benefits from UWV the duration was limited to 36 months. The amount of the funding (including pay for performance) was higher for clients who received social assistance benefits than for clients who received disability benefits. All the IPS intakes were funded by the health insurance company for a maximum of 8 h; during this intake, the IPS employment specialist and the client decided whether IPS was the right intervention for the client [19].

A recent qualitative study among the aforementioned stakeholders has concluded that the collaboration and funding regarding IPS can be improved by applying the multifaceted implementation strategy [19]. However, this study has identified several other barriers that need to be addressed to further improve IPS implementation in practice, such as the temporary and fragmented character of the IPS funding [19].

While several previous studies have focused on clients’ [5, 24–26] or employment specialists’ [27, 28] experiences with IPS, no research has been conducted on clients’ and employment specialists’ experiences with IPS + MIS. Although the multifaceted implementation strategy was primarily aimed at professionals involved in IPS, it is valuable to incorporate input from both employment specialists and from clients to advance understanding of how this strategy may improve IPS implementation and employment outcomes.

This qualitative study draws from a larger study investigating the effectiveness of IPS + MIS, which recruited participants at the community mental health care divisions targeted at adults with SMI of the two mental health agencies involved. Inclusion criteria were: having a SMI, age between 18 and 65 years, having participated in IPS + MIS, and being willing to give informed consent. Exclusion criteria were: full-time hospitalisation.

### Aims

1. To explore experiences with IPS + MIS, and obtaining and maintaining competitive employment among IPS clients.
2. To explore experiences with IPS + MIS, and helping clients to obtain and maintain competitive employment among IPS employment specialists.

## Methods

### Study design

A qualitative, exploratory study was performed using semi-structured interviews with IPS clients and focus groups with IPS employment specialists to collect rich information about experiences with IPS + MIS, and competitive employment. The consolidated criteria for reporting qualitative research (COREQ) [29] were used for the study design and reporting.

### Participants

#### IPS clients

IPS clients were selected in this study if they participated in the larger IPS + MIS study (described previously) using purposeful sampling, defined as identifying and selecting information-rich participants that are especially knowledgeable about or experienced with the topics of interest [30]. This procedure was used to increase heterogeneity among the clients and concerned selecting both clients who had been employed and unemployed during their IPS trajectory, according to the data of the IPS + MIS study. Furthermore, heterogeneity was increased by selecting clients based on gender, age, education, type of benefits (social assistance or disability), employment specialist and mental health agency. Eight employed and eight unemployed clients were contacted by telephone and were provided with information about the aim and procedures of the study. Six employed and four unemployed clients were willing to participate. They were sent an information letter about the study by email. The email also included the date and time of the interview, and the remark that the client could contact the researcher (M.V.) by telephone or email if the client had additional questions. Accordingly, ten clients were recruited and interviewed.

#### IPS employment specialists

The employment specialists in this study were part of specialized mental health treatment teams of the two mental health agencies involved in the aforementioned collaboration, and provided IPS services to the intervention participants in the effectiveness study, according to the IPS model [13, 19]. They were supervised by two IPS program leaders. Eligibility criteria were: having completed the training to become an IPS employment specialist, having provided IPS + MIS, and being willing to give informed consent. Purposeful sampling [30] was used to recruit employment specialists via these IPS program leaders. The program leaders were asked to select employment specialists varying in gender, age and years of experience to create heterogeneous focus groups. They were also asked to distribute an information letter about the study to the selected employment specialists. All selected employment specialists (*n* = 15) were willing to participate. Eight employment specialists from the mental health agency involved in the aforementioned collaboration from the beginning, and seven employment specialists from the mental health agency that joined this collaboration later, were recruited for two focus groups.

### Procedures

#### Interviews

The semi-structured interviews (*n* = 10) were conducted between July 2018 and March 2019 by M.V., trained and experienced in qualitative research. Interviews lasted about 1 h (range 36–74 min) and were voice-recorded. All interviews were face to face and took place at the clients’ mental health agency. At the start of the interview, the aim and procedures of the study were explained by the researcher, and the informed consent form was signed. A topic guide with open-ended questions was developed, based on literature [19, 31–33] and extensive discussions during several meetings of the research team. This guide was used for the interviews to ensure comparability of the interviews, increasing reliability. Additional file 1 provides an detailed overview of the interview topics and questions [Additional file 1]. During the interviews, clients were asked to tell the researcher about their experiences with the IPS trajectory and the multifaceted implementation strategy, i.e. IPS funding and the collaboration between their employment specialist and professionals of their benefits agency. Furthermore, they were asked to tell about their experiences with employment. To elicit any information the clients deemed important, open narrations were encouraged. At the end of each interview, the clients received a gift card. After 10 interviews no new themes emerged related to experiences with IPS and the multifaceted implementation strategy; at that point it was concluded that data saturation was achieved [34]. Therefore, no additional interviews were conducted.

#### Focus groups

The two focus groups were conducted in May and June 2019, and took place at the employment specialists’ mental health agencies. Both focus groups lasted about 2.5 h and were voice-recorded. The first focus group was moderated by H.M., trained and experienced in qualitative research; M.V. was present as an observer, assisting the moderator and monitoring the group interaction. M.M. was present as secretary, taking notes. The second focus group was moderated by M.V.; a trained research assistant, working in the field of public and occupational health, was present as an observer, also taking notes. A topic guide with open-ended questions was developed, based on literature [19, 31–33] and extensive discussions during several meetings of the research team. This guide was used to ensure comparability of the focus groups, increasing reliability. At the start of the focus group, the aim and procedures of the study were explained by the moderator, and the informed consent forms were signed. Then, the topics were discussed. Additional file 2 provides a detailed overview of the focus group topics and questions [Additional file 2]. The employment specialists were asked to discuss their experiences with IPS and helping clients obtaining and maintaining competitive employment. Furthermore, they were asked to discuss their experiences with the multifaceted implementation strategy, i.e. IPS funding and the collaboration with professionals of the benefits agencies. The employment specialists received no compensation for their participation in the focus groups. After two focus groups no new themes emerged related to experiences with IPS and the multifaceted implementation strategy [34]; at that point it was concluded that data saturation was achieved. Therefore, no additional focus groups were conducted.

#### Ethical considerations

The Medical Ethics Committee of the VU University Medical Center gave approval for the study. All procedures performed in this study were in accordance with the ethical standards of this institutional research committee and with the 1964 Helsinki declaration and its later amendments or comparable ethical standards. Written informed consent was obtained from all participants included in the study.

### Data analysis

The interviews and focus groups were transcribed verbatim. Atlas.ti software was used to facilitate data management and analysis. A summary of each interview and both focus groups was made by M.V., and sent by email to the participants concerned for a member check to improve the credibility and validity of the data. In the email, the participants were asked to respond if they had any comments with regard to the summary. This email also included the remark that if the participant did not respond, the researcher would conclude that the participant agreed with the summary content. Two clients and two employment specialists responded; all of them agreed with the summary content. Thematic content analysis was used to analyse the data [35]. The first phase of the analytic process included thoroughly reading all transcripts to become familiar with the data. Relevant text parts were coded and a coding scheme was developed. The next phase included examining similarities and discrepancies in the data, and ultimately grouping and combining codes into themes and subthemes in an iterative manner. All transcripts were coded by M.V.; four interviews and one focus group were coded independently by M.V. and M.M. The codes, themes and subthemes identified by these two researchers were discussed in meetings with a third researcher (F.S.) until consensus was reached. In the last phase, the themes and subthemes were refined by M.V. and F.S. The provisional and final results, including representative quotations from the interviews and focus groups to illustrate them, were critically discussed in meetings with all research team members. These quotations were translated from Dutch to English as literally as possible by a native English speaker.

## Results

Participants’ characteristics are presented in Table 1.

**Table 1:** Participants’ characteristics

IPS clients (*n* = 10)	
Sex female (n)	4
Median age in years (range)	40 (27–55)
Psychotic disorder (n)	7
Low and medium level of education (n)	7
Receiving benefits (n)	8
Competitively employed in the past 5 years (n)	7
Currently employed (n)	6
Median working hours/ week (range)	20 (14–40)
IPS employment specialists (*n* = 15)	
Sex female (n)	13
Median age in years (range)	42 (23–62)
Median number of years of experience as employment specialist (range)	2.5 (1–11)

The thematic content analysis resulted in several themes related to experiences with IPS and the multifaceted implementation strategy. Additional file 3 provides an overview of all identified themes and subthemes at the level of IPS client and employment specialist [Additional file 3]; the most discussed or emphasized themes and subthemes are summarized in Table 2 and reported below.

**Table 2:** Overview of identified themes and subthemes at the level of IPS client and employment specialist

1. Experiences with IPS	
1.1 Importance of discussing client’s motivation and motives to work	Requires attention regularly Various motives to work
1.2 Facilitators to obtaining employment	IPS employment specialist’s crucial role Employers’ inclusiveness Client’s relevant work experience, competences and/ or skills^a^
1.3 Barriers to obtaining employment	Financial factors related to client (e.g. fear of financial decline) Disclosure of client’s mental illness to employer Client’s lack of self-confidence and/ or self-esteem^a^
1.4 Facilitators to maintaining employment	Disclosure of client’s impairments and needs towards employer Positive atmosphere and culture within company
1.5 Barriers to maintaining employment	Client’s mental health problems and susceptibility to stress Financial factors related to employer (e.g. low wage)
2. Experiences with multifaceted implementation strategy	
2.1 Facilitators to collaboration between stakeholders	Regular meetings^b^ Committed contact persons within benefits agencies^b^
2.2 Barriers to benefits counselling	Employment specialist’s limited knowledge regarding benefits Long response time of professionals within benefits agencies Complex laws and legislation regarding social security
2.3 Organizational barriers to IPS execution and collaboration between stakeholders	Lack of continuity^b^
2.4 Financial barriers to IPS execution	Inadequate IPS funding^b^ Variation in follow-up support depending on psychiatrist^b^
2.5 Experiences with pay for performance element	Not aware of pay for performance element Not an appropriate incentive Logical that mental health agency receives extra payments^a^ Does not influence employment specialist^b^

^a^Only perceived by clients. ^b^Only perceived by employment specialists

### Experiences with IPS

#### Importance of discussing client’s motivation and motives to work

Discussing the client’s motivation and motives to work during the IPS trajectory was perceived as important by some clients and most employment specialists. They believed it requires attention regularly and mentioned several reasons for regularly discussing motivation and motives to work from the start of the IPS trajectory: 1) motivation to work is the only criterion for participation in IPS and is also a facilitator for employment, 2) it helps to decide together with the client whether IPS is the right intervention for the client, 3) discussing motives is important for setting goals and if the client’s motivation decreases; by referring to the previously discussed motives and focusing on the positive aspects of work, the employment specialist can promote work motivation, 4) motives can change over time.

*ES14: “[ … ] I explain that motivation is the only thing that counts for participation in IPS. I had another client yesterday and he said: [ … ] ‘so if I don't want to, then I don’t have to?’ And then I thought [ … ]: you are in control. I explain and make this clear right from the start so that they also know that if they are not motivated, they can express that and that they don’t have to think: I am now obliged to participate.”*

Various client’s motives to work were mentioned by both clients and employment specialists. According to the employment specialists and several clients, financial factors, such as low pay and fear of losing benefits, were important for the client and influenced their motivation, choices regarding work and (mental) health.

*Man, employed 24h/w: “It isn’t anything more than benefits, so in that sense, I wouldn’t notice much financially. But yes, if you worked there for five days, you’d have the feeling that you didn’t earn much, and then receiving benefits would almost be more interesting [ … ]. Yes, that might be a reason to leave in the long run, but then of course I’d have to find something that really pays well and suits better.”*

Being occupied (with something meaningful) and a sense of belonging and participation were mentioned as the most important motives to work by most clients.

#### Facilitators to obtaining employment

Both clients and employment specialists mainly mentioned facilitators related to the employment specialist, and reported that the employment specialist had a crucial role during the IPS trajectory. Creating opportunities for the client to gain work and learning experiences, while providing hope and respecting the client, was seen as the employment specialist’s most important task. Meeting the client’s needs and wishes by the employment specialist, and the employment specialist’s involvement and availability were seen as the most important facilitators to obtaining employment.

*Man, employed 26h/w: “[ … ] what do you want yourself? What do you want to achieve? What are your ambitions? She [his IPS employment specialist] started to ask those questions and that is how we came up with what I wanted. [ … ] I am very satisfied with my IPS employment specialist. She did a good job. I'm pretty much where I should be now, and yes, she listened to me and understood my ambitions correctly.”*

Other important facilitators mentioned by both clients and employment specialists, were the employment specialist’s network, and activating and motivating the client by the employment specialist.

*Woman, employed 18h/w: “[ … ] I wasn’t that motivated to start working again. [ … ] Here you were encouraged, like: try it and look how it goes [ … ]. But also: just come every week to our appointment, then we are going to search for a job together. I was just anxious to start working again. [ … ] And here I was told: ‘you can do it, it is going to be alright.’”*

Several employment specialists also pointed out that they have an important task in challenging stigmatizing attitudes among employers and the mental health care providers within their own team, who often underestimate the client’s capabilities to work.

*ES14: “They [her multidisciplinary treatment team] gave me the feedback that they expected me to focus on destigmatization. So, for example, if choices were made about daytime activities or IPS, with new clients, that I would think about those kinds of decisions together with them [her multidisciplinary treatment team] [ … ].”*

Most clients and employment specialists also stated that the employer’s inclusiveness (i.e., a positive attitude towards providing opportunities to people with a mental illness and hiring them) was an important facilitator; according to these employment specialists, inclusive employers often represent small companies that have affinity with people with mental health issues.

Other facilitators mentioned by several clients were relevant work experience, competences and/ or skills of the client.

#### Barriers to obtaining employment

Most clients and employment specialists recognized that financial factors, such as fear of financial decline and a lack of financial incentive, were an important barrier to obtaining employment.

*Woman, unemployed: “[ … ] I thought [ … ] that I would earn less in terms of salary for a job than my benefits. And yes, I thought: well … I’ve already completed my studies and I do want to be rewarded for that.”*

In addition, disclosure of the client’s mental illness towards the employer was considered a barrier. According to several clients and employment specialists, disclosure of the diagnosis can lead to stigma and discrimination, resulting in not being hired. Most of these employment specialists recommended to disclose only relevant information, such as the client’s needs in order to function optimally at work.

*ES9: “In this, I am sometimes directing, well I mean: not directing but more advising. If people do want to disclose and simply say: ‘I have schizophrenia and I have experienced psychoses’, I will say: do you understand that statements like that can evoke certain ideas in a person? Does it serve you to use those terms or can you perhaps use a different way to describe the situation? It often just doesn’t have a positive effect, because people have certain ideas about those terms.”*

Other important barriers mentioned by several clients were the client’s lack of self-confidence and/ or self-esteem, due to a lack of societal participation and a significant distance from the labour market.

#### Facilitators to maintaining employment

Disclosure of client’s impairments and needs towards the employer was considered an important facilitator to maintaining employment by most clients and employment specialists, because it can help create understanding and commitment of the employer and the work environment, and if necessary it may result in work adjustments. They stated it can also create space and reduce stress for the client.

*Woman, employed 18h/w: “I am very sensitive to stress and I don't mind my employer knowing that. When my employer knows what kind of person I am and how I should be treated, it gives me a certain reassurance. It also takes some sort of pressure off [ … ].”*

The other facilitators mentioned were mainly related to the employer and work environment. A positive atmosphere and culture within the company, characterized by a supportive and flexible environment, and opportunities for self-development, were seen as one of the most important facilitators by both clients and employment specialists.

*Woman, employed 20h/w: “I can just be myself with all the things I blurt out [...]. It feels familiar, they [her colleagues] are my type of people [ … ] and it is not much of a business world, [ … ] they are very gentle people and everything just goes the way it goes [ … ]. It is not all very tight, because then I wouldn’t have been able to cope with it [ … ]. I also dare to say what is and what is not going well. I also feel comfortable to say if I have not got around to doing something, I also dare to say what I am unsure about [ … ]; I dare to say all that, to everyone who works there."*

In addition, the presence of an in-company job coach or another supporting professional (e.g. team leader) within the company, was experienced as another important facilitator by several employed clients.

#### Barriers to maintaining employment

Several clients, most of whom were unemployed, and employment specialists indicated that the client’s mental health problems and susceptibility to stress were important barriers to maintaining employment.

*Man, unemployed: “I’m quitting [the job] because I don’t think it is worth being admitted in the clinic for the third time.”*

Several clients and employment specialists also reported that financial factors, such as a low wage, were an important barrier. In addition, financial motives of employers to hire the client were considered a barrier by several employment specialists. They believed that hiring the client because of financial incentives lowers the chance of successfully maintaining a job, as some employers end the client’s contract as soon as they stop meeting the conditions for the financial compensation.

*ES13: “I think that it is important to really check, at an earlier phase, whether an employer is motivated to deal with any difficulties that might exist. If there is only a financial incentive, the chances are that it will fail.”*

### Experiences with multifaceted implementation strategy

#### Facilitators to collaboration between stakeholders

Most clients were not aware that there was a collaboration between their mental health care agency and benefits agency, but they did feel positively about it, once this organizational part of the multifaceted implementation strategy was explained to them again.

The employment specialists felt their collaboration with the professionals of the benefits agencies was improved due to the regular meetings and designated contact persons. According to the employment specialists, these committed contact persons were easy to reach and mainly helped the employment specialists to answer general questions about the client’s benefits, and to refer them to other professionals within the benefits agency who could help the client.

#### Barriers to improving benefits counselling

Despite the regular meetings with the designated contact persons within the benefits agencies, most clients and employment specialists agreed benefits counselling required more attention. In addition, helping clients with their benefits issues was perceived as an important part of the employment specialist’s job. The limited knowledge of the employment specialist regarding benefits issues and the long response time of professionals within the benefits agencies were seen as barriers. Most clients and employment specialists also pointed out that the Dutch laws and legislation regarding social security are complex, making it very difficult to figure out what the financial consequences would be if a benefit recipient started working in a paid job. According to the employment specialists and some clients, this insecurity regarding income consequences of employment resulted in distress in the client and was often a reason for the client to reject activities related to paid employment.

*Woman, employed 20h/w: “[ … ] I was very scared of the financial consequences, because I thought I would really be worse off, financially. That is entirely possible, you could just lose hundreds of euros if you were to start working more. And, no one could tell me where I could find that information; not on the internet, nobody. I called UWV, nobody could tell me … those IPS employment specialists couldn't do that either. I was really worried about that, I was completely stressed.”*

#### Organizational barriers to IPS execution and collaboration between stakeholders

The employment specialists also mentioned some important organizational barriers to the execution of IPS and the collaboration between stakeholders. Lack of continuity due to a high staff turnover and lack of knowledge in newly hired staff was seen as the most important organizational barrier.

*R9: “What affects our results and the way we work with IPS is that for the last three or four years, we’ve had an outflow of IPS employment specialists and then a new inflow and sometimes, for months, no IPS employment specialist in the team, and then once again, someone new that has no experience. [...] You really have to work two years or three years to really improve results and get more people [clients] to work. Plus the number of agreements and contracts that are there, with the municipality, with UWV, that are not always [ … ] transferred properly because there is such an amount of information that people [employment specialists] already have to absorb.”*

In addition, the employment specialists reported that many colleagues felt they were underpaid for their job compared to other mental health care professionals and that this was an important reason for many employment specialists to search for another, better paid job, resulting in a high turnover of employment specialists.

#### Financial barriers to IPS execution

Although most clients did know their IPS trajectory was funded by their benefits agency, they did not mention any barriers related to the IPS funding. The employment specialists, however, agreed that the current IPS funding is inadequate. According to them, the duration of the funding is too short and the amount is too low. Furthermore, they experienced it as a barrier that clients can qualify for funding only once and only for a restricted time period, considering the vulnerability of people with a severe mental illness, with a considerable risk of relapse and losing their job. The lack of possibilities to offer (short-term) follow-up support to clients at risk of losing their job, after the ending of the IPS trajectory, was also seen as an important barrier.

*R13: “[ … ] I now have the first people for whom the IPS trajectory has ended and for whom it’s gone wrong afterwards, and I think it would be very good if there were some kind of fallback possibility. So that in principle, the trajectory does stop, but if it is necessary that you can jump in quickly for a short period.”*

The employment specialists stated that they did try to provide follow-up support, despite the lack of IPS funding. Several employment specialists claimed expenses from the health insurance company for the provided follow-up support, after the ending of the trajectory. To claim these expenses, they needed permission from the psychiatrist. According to the employment specialists, this can result in variation of the support offered depending on the psychiatrist, as some psychiatrists experienced pressure from the health insurer and did not allow this funding to be used for IPS, while other psychiatrists perceived IPS as a part of the mental health treatment and gave permission to claim expenses.

#### Experiences with pay for performance element

Most clients and employment specialists were not aware of the pay for performance element and did not know what happened with the extra payments.

Although most clients felt it was logical that the mental health agency received extra payments when a client was successfully placed in a paid job, a few clients stated it was not appropriate.

*Woman, employed 20h/w: “Isn't the IPS employment specialist getting his salary paid [ … ]? So why should the mental health agency still receive a bonus? I do not really find it necessary [ … ].”*

The employment specialists did not feel a financial incentive, extra motivation or pressure due to the pay for performance element. In addition, they were not aware of the criteria for the extra payments. Some employment specialists thought a financial incentive was not appropriate considering the risk of selection of promising clients; others felt more appreciation and less workload for the employment specialist would be preferable and more motivating for the employment specialist than the pay for performance element.

## Discussion

The aim of the present study was to explore experiences with IPS + MIS, and competitive employment, among IPS clients and employment specialists. Several themes related to experiences with IPS were identified, including the importance of discussing the client’s motivation and motives to work, and facilitators and barriers to obtaining and maintaining employment. Furthermore, several themes related to the multifaceted implementation strategy were identified, including facilitators to collaboration between stakeholders, barriers to benefits counselling, organizational barriers to IPS execution and collaboration between stakeholders, financial barriers to IPS execution and experiences with the pay for performance element. Clients and employment specialists generally had comparable experiences with IPS, implemented by applying the multifaceted strategy, and often mentioned the same facilitators and barriers to obtaining and maintaining employment.

### Comparison with literature

This study found that discussing the client’s motivation and motives to work regularly during the IPS trajectory is important, as clients have various motives to work and their level of motivation and motives can change over time. These findings are in line with previous studies, stressing the importance of addressing motivation to work in people with SMI [36–38].

Another finding in the present study is that the employment specialist’s role was perceived as crucial in helping clients to obtain employment, by both clients and employment specialists; important facilitators were meeting the client’s needs and wishes by the employment specialist, and the employment specialist’s involvement and availability. Existing research examining experiences with IPS reports similar findings [26, 27]. Unlike the present study, these studies [26, 27] focused only on the IPS clients’ perspectives and did not include the employment specialists.

The role of the employment specialist seems less prominent in helping clients to maintain employment, as the most discussed facilitators to maintaining employment in the present study were directly or indirectly related to the employer and the work environment. This limited role of the employment specialist in helping clients to keep their job was also reported by Koletsi et al. [26], who found that IPS clients did not receive as much support from their employment specialist while at work as they would like to have. A more pro-active role of the employment specialist when the client is employed, including more frequent contacts with the client at the job site, may help the client to stay longer employed [39].

An important finding was that the client’s disclosure towards the employer was experienced as both a facilitator and barrier to employment, depending on the timing and the type of information disclosed. Disclosure of the client’s mental illness, e.g. the diagnosis, towards the employer was considered a barrier to obtaining employment, as it can lead to stigma and discrimination, resulting in the client not being hired; disclosure of the client’s work impairments and needs was considered a facilitator to maintaining employment because it can help create understanding and commitment of the employer and the work environment, and if necessary may result in work adjustments. These findings are consistent with those of Brouwers et al. [40], who found that disclosure can lead to stigma and discrimination, but also to work environment support. These findings also suggest that disclosure during the hiring period may better be avoided [40, 41]; once the client is hired, however, disclosure of the client’s work impairments and needs may enhance sustainable employment.

### Strengths and limitations

A strength of this study is that it is the first study exploring experiences with IPS + MIS, and barriers and facilitators to successfully obtaining and maintaining competitive employment, among IPS clients and employment specialists. Another strength is that the COREQ checklist was used for the study design and reporting, improving the quality of this qualitative study [29]. Although qualitative studies tend to have small sample sizes, which may limit generalizability, they can provide more insight.

Purposive sampling was used to increase heterogeneity among the clients and employment specialists, but it was not fully successful, as more included clients were employed than not; it is possible that they had mostly positive experiences with IPS and employment, causing selection bias. In addition, the majority of the included employment specialists was female. Although more differentiation in gender of the employment specialists would have been preferable, it is uncertain whether this influenced the results of this study.

Participants were asked to review a summary of their interview or focus group to improve the credibility and validity of the data. The majority of the participants (21 of 25), however, did not respond to the email including this summary. This may have compromised the quality of the data.

The quotations of the participants were translated from Dutch to English as literally as possible by a native English speaker. Although the essence of the quotations has remained the same, it is possible that the English translations of the quotations do not exactly match the original quotations in Dutch.

### Implications for practice and research

Activating and motivating the client by the employment specialist were experienced as important facilitators to obtaining employment. Additional interventions, such as motivational interviewing, can be used by the employment specialist to further enhance the client’s motivation [36, 37]. This use of additional interventions is likely to reinforce the effects of IPS for clients [16, 37, 42, 43]. The finding that the client’s relevant work experience, competences and/ or skills were experienced as facilitators to obtaining employment, also suggests that additional interventions or training for the client during the IPS trajectory may reinforce the effects of IPS [16]. Implementing additional interventions along with IPS, however, is challenging and needs more attention [42].

Some barriers to obtaining employment identified in this study, such as the client’s lack of self-confidence and/ or self-esteem, appear to have psychosocial underpinnings. Although pre-employment assessments are not recommended in IPS programmes [13], this finding suggests that assessing psychosocial factors in an early phase of the IPS trajectory may be useful for identifying clients with psychosocial issues who would benefit from additional interventions [43].

Financial factors, such as employer-focused financial incentives, may be a facilitator to obtaining employment for people with mental illness [40]. In the present study, however, financial factors were experienced as both a barrier to obtaining and maintaining employment. Financial motives of the employer to hire the client and a low wage for the client, for example, were considered important barriers to maintaining employment. These findings suggest that the current financial incentives for employers and clients should be evaluated and adjusted, as they do not seem to contribute to sustainable employment of clients.

An interesting finding was that both IPS clients and employment specialists were not fully aware of all aspects of the multifaceted implementation strategy. For example, most clients and employment specialists were not aware of the pay for performance element and did not know what happened with the extra payments. This finding suggests that information transfer with regard to the multifaceted implementation strategy needs more attention [19]. Although the multifaceted implementation strategy is primarily aimed at the professionals involved in IPS, the clients should also be informed correctly about the services they are receiving [13, 44], as the pay for performance element, for example, might unintendedly result in adverse client selection and pressure to achieve job placements quickly by the employment specialists [19, 45]. Moreover, helping clients to have a more comprehensive understanding of their IPS trajectory, will foster shared decision making and their self-determination [13, 44].

Although the collaboration between stakeholders and IPS funding seems to be improved due to the multifaceted implementation strategy [19], organizational and financial barriers were mainly identified in the present study. An important theme related to the multifaceted implementation strategy, were barriers to benefits counselling. The limited knowledge of the employment specialist regarding benefits issues was perceived as a barrier, while helping clients with their benefits issues was perceived as an important part of the employment specialist’s job. Furthermore, the Dutch laws and legislation regarding social security were considered complex, making it very difficult to figure out what the financial consequences would be if a benefit recipient started working in a paid job. These findings suggest that employment specialists should receive a targeted training on the relevant, Dutch laws and legislation regarding social security. In addition, the availability of professionals within benefits agencies should be improved, as the long response time of the professionals within the benefits agencies was experienced as a barrier. Clear and accurate information about the impact of having paid employment on the client’s benefits should be readily available and offered to clients, as insecurity regarding income is likely to result in distress and a disincentive to engage in employment [46].

Another important theme related to the multifaceted implementation strategy, were financial barriers to IPS execution. The finding that the lack of possibilities for the employment specialist to offer follow-up support to clients was perceived as an important barrier, suggests that the current IPS funding is still not adequate, as it does not support the key IPS principle of providing time-unlimited, individualised support [13]. An interesting finding is that some psychiatrists who perceived IPS as a part of the mental health treatment, gave permission to employment specialists to claim expenses from the health insurance company for follow-up support, while other psychiatrists did not, resulting in variation of the support for clients depending on the psychiatrist. Currently, only the IPS intake is funded by the health insurance company for a maximum of 8 h [19]. To avoid variation in the follow-up support, as reported in this study, the part of the IPS funding reimbursed by the health insurance company should be adapted to the current practice and formalized [22].

## Conclusions

This qualitative study provides more insight into the IPS clients’ and employment specialists’ experiences with IPS + MIS, and competitive employment. Although the multifaceted implementation strategy seems to contribute to an improved IPS implementation, the barriers identified in this study suggest that further steps are necessary to promote IPS execution and to help people with SMI obtain and maintain competitive employment.

## Supplementary Information

**Additional file 1:.** Experiences with Individual Placement and Support and employment – a qualitative study among clients and employment specialists. Overview of the interview topics and questions.

**Additional file 2:.** Experiences with Individual Placement and Support and employment – a qualitative study among clients and employment specialists. Overview of the focus group topics and questions.

**Additional file 3:.** Experiences with Individual Placement and Support and employment – a qualitative study among clients and employment specialists. Overview of all identified themes and subthemes at the level of IPS client and employment specialist.

## Data Availability

The datasets used and/ or analysed during the current study are available from the corresponding author on reasonable request.

## Abbreviations

IPS: Individual placement and support
SMI: Severe mental illness
IPS + MIS: Individual placement and support using a multifaceted implementation strategy
COREQ: Consolidated criteria for reporting qualitative research
UWV: The dutch social security institute: the institute for employee benefits schemes
