# Supplementary material for: Experiences with Individual Placement and Support and employment – a qualitative study among clients and employment specialists
Source: BMC Psychiatry. 2021 Apr 7;21:181. doi: 10.1186/s12888-021-03178-2 (PMC8025385; doi:10.1186/s12888-021-03178-2)
Supplement: Supplementary file 1 — Additional file 1:. Experiences with Individual Placement and Support and employment – a qualitative study among clients and employment specialists. Overview of the interview topics and questions. [file 12888_2021_3178_MOESM1_ESM.docx]

**Additional file 1: Experiences with Individual Placement and Support and employment – a qualitative study among clients and employment specialists**

**Overview of the interview topics and questions**

General information about the client

Current age

Diagnosis

Level of education

Current situation regarding benefits

Current situation regarding the IPS trajectory

Competitively employed in the past 5 years (yes or no)

Current situation regarding competitive employment

IPS trajectory

What are reasons or motives for you to work?

What are reasons or motives for you to start with IPS?

What are your experiences with your IPS trajectory?

What kind of support or help did you receive?

What is going well? What are you satisfied with?

What is not going well? What are you not satisfied with?

Do you have any suggestions to improve the IPS trajectory?

How is the relationship with your IPS coach? How did you experience the role of the IPS coach?

Has your mental health care provider had a role during your IPS trajectory? If so, what was the role of your mental health care provider(s)?

What are your experiences with the collaboration between your employment specialists and your mental health care provider(s)? What is your opinion on this collaboration?

Multifaceted implementation strategy

Have professionals of your benefits agency had a role during your IPS trajectory? If so, what was the role of these professionals?

Has your health insurance company had a role during your IPS trajectory? If so, what was the role of your health insurance company?

What are your experiences with the collaboration between your employment specialist and the professionals of your benefits agency regarding your IPS trajectory? What is your opinion on this collaboration?

What are your experiences with the IPS funding? Do you know who finances your IPS trajectory? Do you know that your mental health agency receives extra payments when your employment specialists places you in a competitive job? What is your opinion on this?

Obtaining and maintaining employment

What are your experiences with employment?

What helps/ helped you to obtain a job? According to you, what are/ were facilitators?

What helps/ helped you to continue working in a job? According to you, what are/ were facilitators?

Which barriers to obtaining a job did you experience?

Which barriers to maintaining a job did you experience?

Can you give any reasons why you have not been successful in obtaining and/ or maintaining a job (so far)?

How do you experience your current job? What is going well? Are you satisfied? What is not going well? What do you need to improve your situation?

What is your opinion on disclosure of your mental illness towards your (future) employer? What are your experiences with disclosure so far?

According to you, what are the effects of employment or employment related activities on your health and daily functioning?
