# Supplementary material for: Experiences with Individual Placement and Support and employment – a qualitative study among clients and employment specialists
Source: BMC Psychiatry. 2021 Apr 7;21:181. doi: 10.1186/s12888-021-03178-2 (PMC8025385; doi:10.1186/s12888-021-03178-2)
Supplement: Supplementary file 2 — Additional file 2:. Experiences with Individual Placement and Support and employment – a qualitative study among clients and employment specialists. Overview of the focus group topics and questions. [file 12888_2021_3178_MOESM2_ESM.docx]

**Additional file 2: Experiences with Individual Placement and Support and employment – a qualitative study among clients and employment specialists**

**Overview of the focus group topics and questions**

General information about the employment specialist

Current age

Number of years of experience as IPS employment specialist

IPS trajectory

- Client’s motivation and motives to work and to start with IPS

What role does motivation play during the IPS trajectory?

Do you ask clients what their motives are to work? If so, why?

- Experiences with IPS and helping clients to obtain and maintain employment

How do you perceive your role as IPS employment specialist?

What do you do to help clients to obtain employment? What is going well? What are points for improvement? According to you, what are facilitators and barriers to obtaining employment for clients?

What do you do to help clients to maintain employment? What is going well? What are points for improvement?

According to you, what are facilitators and barriers to maintaining employment for clients?

According to you, what is going well within the IPS trajectory? What are points for improvement?

What is your opinion on disclosure of the client’s mental illness towards the employer? What are the benefits? What are the disadvantages?

According to you, what are the effects of employment or employment related activities on the client’s health and daily functioning?

- Collaboration between employment specialists and mental health care providers

How do you see your role within your multidisciplinary treatment team? How are you seen by the other members of your team?

How do you experience your collaboration with the mental health care providers within your team? What is going well? What are points for improvement?

What is the role of the mental health care providers during the IPS trajectory? What is going well? What are points for improvement?

Multifaceted implementation strategy

- Collaboration between employment specialists and professionals of the benefits agencies

How do you experience your collaboration with professionals of the benefits agencies? What is going well? What are points for improvement?

- IPS funding

What do you think of the current IPS funding? How satisfied are you with the IPS funding? What are points for improvement?

What is your opinion on the pay for performance element?
