# Supplementary material for: Experiences with Individual Placement and Support and employment – a qualitative study among clients and employment specialists
Source: BMC Psychiatry. 2021 Apr 7;21:181. doi: 10.1186/s12888-021-03178-2 (PMC8025385; doi:10.1186/s12888-021-03178-2)
Supplement: Supplementary file 3 — Additional file 3:. Experiences with Individual Placement and Support and employment – a qualitative study among clients and employment specialists. Overview of all identified themes and subthemes at the level of IPS client and employment specialist. [file 12888_2021_3178_MOESM3_ESM.docx]

**Additional file 3: Experiences with Individual Placement and Support and employment – a qualitative study among clients and employment specialists**

| Overview of all identified themes and subthemes at the level of IPS client and employment specialist | |
| --- | --- |
| 1. Experiences with IPS |  |
| 1.1 Importance of discussing client’s motivation and motives to work | Requires attention regularly  Various motives to work  Financial factors  Being occupied (with something meaningful)  Sense of belonging and participation  Structure  Self-development  Promoting recovery  Improving self-esteem  Having a purpose  Social contacts^b^ |
| 1.2 Facilitators to obtaining employment | IPS employment specialist’s crucial role  Creating opportunities for client to gain work  Providing hope and respecting client  Meeting client’s needs and wishes  Being involved and available  Having a network  Activating and motivating client  Destigmatizing^b^  Employers’ inclusiveness  Client’s relevant work experience, competences and/ or skills^a^  Support from client’s family and friends^a^  Mental health care provider providing adequate and needed care |
| 1.3 Barriers to obtaining employment | Financial factors related to client  Fear of losing benefits or issues with benefits  Fear of financial decline  Lack of financial incentive  Disclosure of client’s mental illness to employer  Client’s lack of self-confidence and/ or self-esteem^a^  Mental health care provider not referring client to IPS^b^  Financial factors related to employer  Low wage  No travel allowance^b^  Long distance to work^a^ |
| 1.4 Facilitators to maintaining employment | Disclosure of client’s impairments and needs towards employer  Positive atmosphere and culture within company  Mental health care provider providing adequate and needed care Support from client’s family and friends  Client’s wish for self-development^a^ |
| 1.5 Barriers to maintaining employment | Client’s mental health problems and susceptibility to stress  Financial factors related to employer  Low wage  Issues with payment of wage^b^  Financial motives to hire client^b^  Organizational issues within company |
| 1.6 Positive effects of employment (related activities) on health and functioning | Less health care consumption  Feeling of recovery  Structure  Self-development  Improved self-confidence and/ or self-esteem |
| 1.7 Negative effects of employment (related activities) on health and functioning | Feeling stressed and insecure (about financial situation)  Relapse of mental illness  Work related physical complaints^a^  Relational problems^a^ |
| 1. Experiences with multifaceted implementation strategy |  |
| 2.1 Facilitators to collaboration between stakeholders | Regular meetings^b^  Committed contact persons within benefits agencies^b^ |
| 2.2 Barriers to benefits counselling | Employment specialist’s limited knowledge regarding benefits  Long response time of professionals within benefits agencies  Complex laws and legislation regarding social security |
| 2.3 Organizational barriers to IPS execution and collaboration between stakeholders | Lack of continuity^b^  Lack of staff capacity^b^  High work load for mental health care staff^b^ |
| 2.4 Financial barriers to IPS execution | Inadequate IPS funding^b^  Variation in follow-up support depending on psychiatrist^b^ |
| 2.5 Experiences with pay for performance element | Not aware of pay for performance element  Not an appropriate incentive  Logical that mental health agency receives extra payments^a^  Does not influence employment specialist^b^ |

^a^Only perceived by clients. ^b^Only perceived by employment specialists.
